# Correction: Thermally stable mesoporous tetragonal zirconia through surfactant-controlled synthesis and Si-stabilization

**DOI:** 10.1039/d2ra90091b

**Published:** 2022-09-15

**Authors:** Ken L. Abel, Sebastian Weber, David Poppitz, Juliane Titus, Thomas L. Sheppard, Roger Gläser

**Affiliations:** Institute of Chemical Technology, Universität Leipzig 04299 Leipzig Germany roger.glaeser@uni-leipzig.de; Institute for Chemical Technology and Polymer Chemistry, KIT (Karlsruhe Institute of Technology) 76131 Karlsruhe Germany; Institute of Catalysis Research and Technology, Karlsruhe Institute of Technology (KIT) 76344 Eggenstein-Leopoldshafen Germany

## Abstract

Correction for ‘Thermally stable mesoporous tetragonal zirconia through surfactant-controlled synthesis and Si-stabilization’ by Ken L. Abel *et al.*, *RSC Adv.*, 2022, **12**, 16875–16885, https://doi.org/10.1039/d2ra01459a.

The authors regret that an incorrect version of Table 2 was included in the original article. The correct version of Table 2 is presented below.

**Table:** Weight fraction of t-ZrO_2_ (*ω*_t-ZrO_2__) and mean crystallite size (*d*_c_) for zirconia samples calcined at 973 K with different mole fractions of Si (*y*S), prepared in the presence of 20 mol% DDA during gelation (−20D). Values and error ranges calculated from Rietveld refinement. For detailed information, see section S3.1 in the ESI

Sample	*ω*_t-ZrO_2__/wt%	*d*_c_/nm
0SZ-20D	4.98(4)	25.03(12)
5SZ-20D	38.08(11)	14.6(2)
10SZ-20D	100	11.87(14)
15SZ-20D	100	8.64(10)
29SZ-20D	100	8.10(12)
40SZ-20D	n.a.^a^	n.a.^a^

a Not applicable.

The Royal Society of Chemistry apologises for these errors and any consequent inconvenience to authors and readers.

## Supplementary Material

